# Clinical Outcomes and Cost-Effectiveness Analysis of FFR Compared with Angiography in Multivessel Disease Patient

**DOI:** 10.5935/abc.20180262

**Published:** 2019-01

**Authors:** Edgard Freitas Quintella, Esmeralci Ferreira, Vitor Manuel Pereira Azevedo, Denizar V. Araujo, Fernando Mendes Sant'Anna, Bernardo Amorim, Denilson Campos de Albuquerque

**Affiliations:** 1 Hospital Universitário Pedro Ernesto - Universidade Estadual do Rio de Janeiro (UERJ), Rio de Janeiro, RJ - Brazil; 2 Instituto Estadual de Cardiologia Aloysio de Castro (IECAC), Rio de Janeiro, RJ - Brazil; 3 Cardiology Department. Hospital Clínico Universitario, INCLIVA. Universitat de València, Valencia - Spain; 4 Instituto Nacional de Cardiologia Laranjeiras (INCL), Rio de Janeiro, RJ - Brazil

**Keywords:** Fractional Flow Reserve, Myocardial, Cost-Benefit Analysis, Coronary Artery Disease/economics, Angioplasty, Balloon, Coronary, Stents

## Abstract

**Background:**

In multivessel disease patients with moderate stenosis, fractional flow reserve (FFR) allows the analysis of the lesions and guides treatment, and could contribute to the cost-effectiveness (CE) of non-pharmacological stents (NPS).

**Objectives:**

To evaluate CE and clinical impact of FFR-guided versus angiography-guided angioplasty (ANGIO) in multivessel patients using NPS.

**Methods:**

Multivessel disease patients were prospectively randomized to FFR or ANGIO groups during a 5 year-period and followed for < 12 months. Outcomes measures were major adverse cardiac events (MACE), restenosis and CE.

**Results:**

We studied 69 patients, 47 (68.1%) men, aged 62.0 ± 9.0 years, 34 (49.2%) in FFR group and 53 (50.7%) in ANGIO group, with stable angina or acute coronary syndrome. In FFR, there were 26 patients with biarterial disease (76.5%) and 8 (23.5%) with triarterial disease, and in ANGIO, 24 (68.6%) with biarterial and 11 (31.4%) with triarterial disease. Twelve MACEs were observed - 3 deaths: 2 (5.8%) in FFR and 1 (2.8%) in ANGIO, 9 (13.0%) angina: 4(11.7%) in FFR and 5(14.2%) in ANGIO, 6 restenosis: 2(5.8%) in FFR and 4 (11.4%) in ANGIO. Angiography detected 87(53.0%) lesions in FFR, 39(23.7%) with PCI and 48(29.3%) with medical treatment; and 77 (47.0%) lesions in ANGIO, all treated with angioplasty. Thirty-nine (33.3%) stents were registered in FFR (0.45 ± 0.50 stents/lesion) and 78 (1.05 ± 0.22 stents/lesion) in ANGIO (p = 0.0001), 51.4% greater in ANGIO than FFR. CE analysis revealed a cost of BRL 5,045.97 BRL 5,430.60 in ANGIO and FFR, respectively. The difference of effectiveness was of 1.82%.

**Conclusion:**

FFR reduced the number of lesions treated and stents, and the need for target-lesion revascularization, with a CE comparable with that of angiography.

## Introduction

In stable coronary artery disease (CAD), angiographic lesions that would benefit most from myocardial revascularization (MR) are those associated with ischemia.^1^

Non-invasive tests (NITs) for ischemia may yield conflicting results, which make it difficult to identify culprit lesions based on symptoms, and consequently to make better therapeutic decisions.^2^ In multivessel coronary disease patients, angiography may fail to evaluate the prognosis, especially in those with moderate stenosis (50-70%).^3^

FAME-2^4^ trial compared the use of fractional flow reserve (FFR) and angiography alone to identify coronary stenosis that required treatment. The study could be discontinued earlier due to the superiority of FFR-guided revascularization.

Although most percutaneous coronary interventions (PCIs) are still performed without NITs, 70% of patients referred for PCI have multivessel diseases, and 80% have moderate lesions.^5^ However, it is estimated that 40-50% of these lesions are ischemic.

FFR is the best method to associate obstruction with ischemia. A FFR < 0.75 is considered to be associated with ischemia, with sensitivity, specificity, positive and negative predictive values greater than 90%.^6^^,^^7^ PCI for ischemic lesions is cost-effective and decreases the occurrence of major adverse cardiac events (MACE).^8^

Fearon et al.^9^ showed that FFR-guided PCI in patients with one-vessel CAD was superior to other therapeutic strategies based on angiography or scintigraphy.

Our study aims to add to the knowledge of the cost-effectiveness (CE) of FFR-guided PCI in patients with multivessel CAD.

## Objectives

To assess the occurrence of MACE and CE of FFR, compared with angiographic criteria for patients with multivessel diseases undergoing PCI.

## Methods

Prospective, randomized, clinical study on PCI in 70 patients with multivessel disease attending Pedro Ernesto University Hospital of the Federal University of Rio de Janeiro and Aloysio Castro Institute of Cardiology between April 2011 and May 2016.

Patients were randomized using computer-generated random numbers (R software, 2.11) to:

1. FFR measurements of significant lesions and PCI with stent implantation for lesions with FFR < 0.75 (FFR group);
2. PCI with stent implantation for stenosis > 60% by visual assessment with angiography (ANGIO group).

Each computer-generated number corresponded to one group. The numbers were put into opaque, sealed envelopes, which were sequentially opened for each patient recruited for the study by an independent person who was unaware of the allocation.

Sample size was calculated using Epi-Info software, version 3.4, considering a power (1-β) of 80% and 95% confidence interval. An estimated 17% difference in the costs between the two groups was used for calculation of the sample size required to reach statistically significant difference.

The sample size calculated was 200 (100 for each group); however, due to financial constraints, the number of patients included was 70.

### Population

Patients aged 21 years or older with stable multivessel disease or at day 7 after acute coronary syndrome (ACS), with at least one moderate stenosis (>60%) without severe left ventricular dysfunction, and with NIT for ischemia, were divided into two groups (Table 1). In group 1 (FFR, n = 34), PCI was performed for FFR < 0.75, whereas in group 2 (ANGIO, n = 35), patients underwent PCI with stent implantation in all significant lesions. One patient was lost to follow-up, and a total of 69 patients were studied. Dual antiplatelet therapy (DAPT) was used for at least 6 months. Patients were assessed at 30 days, six months and one year of follow-up (Table 2). At six months, NIT and coronary angiography were performed in symptomatic or ischemic patients; FFR measurements were performed again in the first group, and restenosis was treated according to the course of disease.

**Table 1:** Characteristics of the study population (overall and by group)

	Overall study population (%)	FFR n (%)	ANGIO n (%)	p
Number of patients	69 (100.0)	34 (49.3)	35 (50.7)	-
Male sex	47 (68.1)	25 (53.2)	22 (46.8)	0.342*
Female sex	22 (31.9)	9 (40.9)	13 (59.1)	0.342*
Diabetes	24 (35.8)	12 (50.0)	12 (50.0)	0.930*
Hypertension	51 (73.9)	25 (49.0)	26 (50.9)	0.943*
Dyslipidemia	50 (72.5)	24 (42.0)	26 (52.0)	0.731*
Family history	40 (57.9)	21 (52.5)	19 (47.5)	0.529*
Current smoker	19 (27.5)	10 (52.6)	9 (47.4)	0.731*
Previous AMI	15 (21.7)	8 (53.3)	7 (46.7)	0.722*
Stable angina	42 (60.8)	20 (47.6)	22 (52.3)	0.930^‡^
Acute coronary syndrome	27 (39.1)	14 (57.1)	13 (42.8)	0.930^‡^
Age (years) mean ± SD	62.0 ± 9.0	62.7 ± 8.4	59.5 ± 9.4	0.117*
LV ejection fraction (%) (mean ± SD)	67.0 ± 13.3	70.0 ± 14.0	64.0 ± 12.0	0.110^†^

AMI: acute myocardial infarction; FFR: fractional flow reserve group; ANGIO: coronary angiography group; SD: standard deviation; LV: left ventricle.

* Pearson's chi-square test;

† Kruskal-Wallis test;

‡ Fisher's exact test.

**Table 2:** Major adverse cardiovascular events in the study population

	Study population (%)	FFR n (%)	ANGIO n (%)
MACE	12 (17.3)	6 (17.6)	6 (17.1)
Total deaths	3 (4.3)	2 (5.8)	1 (2.8)
Deaths from cardiovascular causes	2 (2.8)	1 (2.9)	1 (2.8)
Deaths from non-cardiovascular causes	1 (1.4)	1 (2.9)	0 (0.0)
Angina	9 (13.0)	4 (11.7)	5 (14.2)
Target lesion revascularization	6 (8.6)	2 (5.8)	4 (11.4)*

FFR: fractional flow reserve group; ANGIO: coronary angiography group; MACE: major adverse cardiovascular events;

* 1 patient missed second coronary angiography and was lost to follow-up.

### Cost-effectiveness

We used the CE model proposed in the Brazilian study by Polanczyk et al.^10^ CE outcome measure was “one-year restenosis-free survival”.

### Effectiveness analysis

Estimates were obtained from the literature,^10^ and the cost of procedure index calculated under the perspective of the Brazilian Unified Health System (SUS). We analyzed the mean costs of each intervention, considering SUS’s reimbursement to the hospitals. For each intervention, we calculated expected costs and the clinical outcomes described above.

### Statistical analysis

Data were described as frequency, mean and standard deviations, and median and interquartile ranges. The Kruskal-Wallis test was used for outcome comparisons between the groups, and the Pearson’s chi-square test or Fisher’s exact test was used for comparisons of dichotomous variables. Logistic regression was used to analyze the association between independent variables and outcomes. Kaplan-Meier survival curves were constructed and compared by log-rank test. Survival was analyzed by bivariate and multivariate Cox regression analysis. SATAT 14 (SATA Inc) software was used for analysis. The level of significance was set at p ≤ 0.05%. All tests were two-tailed.

## Results

**Figure 1:**
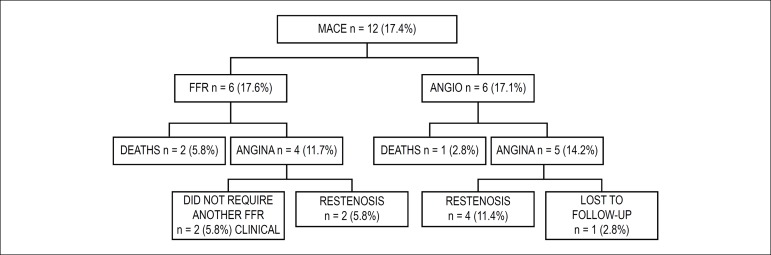
Flowchart of major cardiac events (MACE) by study group. FFR: fractional flow reserve group; ANGIO: coronary angiography group.

Patients’ characteristics are described in Table 1. Most patients had a stable disease, or those with ACS patients were asymptomatic for 7 days. MACEs were reported by 12 patients (17.3%) - 6 patients (17.1%) in FFR group and 6 patients (17.1%) in ANGIO group. Three deaths occurred, 2 (2.8%) in the FFR group and 1 (1.4%) in the ANGIO group (AMI, without DAPT discontinuation). Nine (13.0%) had angina, 4 (5.7%) in FFR group and 5 (7.2%) in the ANGIO group (Figure 1). In the 4 patients of the FFR group, based on FFR measurements, 2 patients did not require a second PCI and continued in medical treatment. In the other 2 patients, intra-stent restenosis was confirmed, and these patients were treated with pharmacological stents (PS), with satisfactory results.

**Figure 2:**
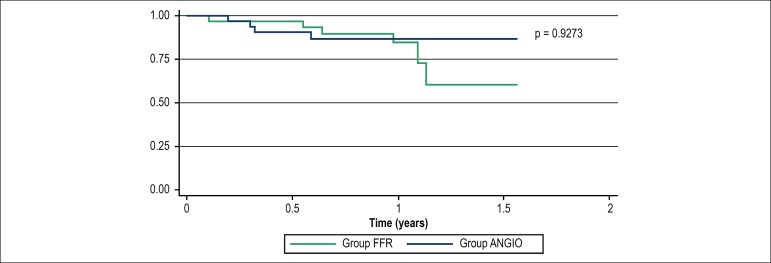
Event-free survival curve (Kaplan-Meier) by group in an 18 month-period FFR: fractional flow reserve group; ANGIO: coronary angiography group.

In group 2, one symptomatic patient with mild apical ischemia (according to scintigraphy), continued on medical treatment despite restenosis of marginal branch, but without restenosis of right coronary artery (Table 3). Event-free survival curve in the study population and by groups during the 18-month period of follow-up is depicted in Figure 2.

**Table 3:** Characteristics of patients with compound events (Angina/Restenosis)

	FFR				Total	ANGIO					Total
Number of patients	1	2	3	4		1	2	3	4	5	
Angina								No			
Asymptomatic / (+) ischemia	No	No	No	No	No	No	No	No	No	No	
Vessels to be treated	2	2	2	2	8	3	2	3	2	2	12
Vessels treated	1	0	1	0	2	3	2	3	2	2	12
Control catheterization									No		
Vessels with restenosis	1	0	1	0	2	2	3	1	(?)	1	7
Target-lesion revascularization	1	0	1	0	2	2	1	No	No	1	4

FFR: fractional flow reserve group; ANGIO: coronary angiography group.

### Angiographic results

In the analysis by group, no difference was observed in the number of lesions evaluated (vessels that require treatment) between the groups. There was a balanced distribution of lesions between anterior descending artery, circumflex artery and right coronary artery.

### Lesions by study group

No difference was found in the number of stents per patient, with a mean of 1.0 ± 0.2 stents per lesion in the ANGIO group, and 0.4 ± 0.5 in the FFR group (p = 0.0001) (Kruskal-Wallis), i.e. a 50% reduction. The number of lesions treated in ANGIO group was 65% greater than in FFR group. On the other hand, 45% of lesions analyzed in FFR were treated. In ANGIO group, stent implantation per patient was more than twice the number observed in FFR group (1.1 vs. 2.2 *stents*/patient). Characteristics of the lesions were assessed by angiographic quantification. In group 1, FFR were measured before and after procedure (Table 4).

**Table 4:** Mean fractional flow reserve before and after percutaneous coronary intervention

	n	FFR (mean ± SD)	p
Before PCI	87	0.74 ± 0.15	0.290*
Post-PCI	39	0.90 ± 0.06	0.290*

FFR: fractional flow reserve; PCI: percutaneous coronary intervention; SD: standard deviation;

* Pearson's chi-square test.

### Cost-effectiveness

Estimates of the main clinical outcomes and probabilities to be included in the decision model were obtained from the literature, by review of randomized trials involving non-pharmacological stents (NPS) and PCI. Procedure-index cost and, the cost of post-PCI stable stage, and other costs were expressed in Brazilian Reals (BRL) (Table 5).^10^ The difference in effectiveness, costs and incremental CE ratio (ICER) were 1.8%, BRL384.61, and BRL21,156.55, respectively (Table 6).

**Table 5:** Estimates for the model: procedure and outpatient service costs

Procedures	Costs (BRL)	
	ANGIO	FFR
Procedure-index	1,503.00	1,503.00^28^
(*Stent* and FFR - mean cost)	2,034.50	2,517.25
Restenosis management (ICP + c/ SF*)	7,904.01^29^	
Revascularization surgery - elective	7,620.60^29^	
- emergency	8,950.50^28^	
AMI-index	2,716.95^29^	
One year without events following ICP or stable MRS	1,383.00^28^	
Cardiac catheterization	539.00^28^	
Mean PCI	5,386.76^29^	
PCI with balloon	1,599.02^29^	
Death for CAD	2,577.00^28^	

PCI: percutaneous coronary intervention; AMI: acute myocardial infarction; CAD: coronary artery disease; MRS: myocardial revascularization surgery; ANGIO: coronary angiography group; FFR: fractional flow reserve group.

* Management of restenosis with percutaneous coronary intervention + covered stent.

**Table 6:** Results of cost-effectiveness analysis: coronary angiography (ANGIO) group versus fractional flow reserve (FFR) group

Strategy	One-year effectiveness	Difference in effectiveness	Cost (BRL)	Cost difference (BRL)	ICER
ANGIO	78.52%	-	5,045.97	-	-
FFR	80.34%	1.82%	5,430.60	384.62	21.156.55

ICER: incremental cost-effectiveness ratio; ANGIO: coronary angiography group; FFR: fractional flow reserve.

## Discussion

The present study shows that FFR-guided PCI is a cost-effective strategy compared with angiographic criteria in patients with multivessel diseases, reducing the number of stenosis, stents and need for target lesion revascularization (TLR).

Asymptomatic patients, even elderly patients older than 75 years,^11^ with percent myocardial ischemia ≥ 10% ischemic benefit from MR. In the COURAGE trial nuclear substudy,^12^ patients that achieved a reduction in ischemic myocardium from ≥10% to <5%, showed better outcomes. Reduction of risk factors is essential in medical therapy. In this regard, to reduce the extension and severity of ischemic myocardium may contribute to the improvement of patients’ quality of life, particularly among those whose medical treatment was shown to be ineffective. The correlation of coronary anatomy with ischemic parameters may provide a rational and safe basis for revascularization. The ISCHEMIA trial,^13^ still under way, was designed to compensate for existing limitations in the literature. In the present study, we attempted to show a reduction in MACE with FFR-guided invasive strategy compared with optimized medical treatment, and only for patients that did not respond to medical treatment.

The key point in performing or not MR is the possibility of quantifying ischemic lesions per segment in case of multiple lesions, especially when associated with moderate lesions, which represent most of the cases. In this context, the only method capable of showing this relationship is FFR. However, the method is not only an invasive strategy, but also involves higher costs. In Brazil, the reality of PCI is very particular. Although their coverage by SUS was approved in August 2014, due to their high costs, PSs are not widely provided by the system. Instead, their use is restricted to diabetic patients in whom vessels with diameter <2.5 mm and extension >18 mm is observed.^14^

The choice to treat with percutaneous revascularization mutivessel diseases was grounded in studies on FS - the SYNTAX,^13^ FAME^15^ and FAME-2^4^ studies.

Data on revascularization with NPS and FFR are scarce. However, the use of FFR in multivessel diseases have been evaluated, with no difference in mortality or non-fatal infarction, despite differences in TLR.^16^

This randomized, prospective study on patients with multivessel diseases referred for FFR- or angiography-guided PCI was based on FAME study,^15^ using NPS though. Also, in our study, lesions with FFR > 0.75 were not treated, different from FAME, that used a cut-off of 0.80. The choice for a lower cut-off point was justified by a 100%^16^ predictive value for a FFR value of 0.75. A cut-off of 0.75 would hence represent a lower chance of restenosis, since it would be expected a higher incidence of restenosis with the use of NPS.

Li et al.^17^ evaluated more than 7,300 patients, 1,090 of them undergoing FFR-guided PCI, 30% with NPS. After the exclusion of patients with FFR > 0.75 and < 0.80, there was a decrease in the rates of AMI and in the composite of AMI and death. In patients with FFR > 0.80, a conservative approach was used.

### Clinical data

Although the increment of 1.45% in mortality in the FFR group was not statistically significant, the result contrasts with the literature, although we attributed this finding to the small number of randomized patients.^17^^-^^19^ Zhang et al.^20^ showed in a meta-analysis including nearly 50,000 patients that FFR reduced the absolute risk of late mortality by 7.7%.^20^

The frequency of MACE in our study group was 17.3%, with similar distribution between the groups, in accordance with the FAME study.^15^ The incidence of angina in the FFR group was identical between the groups.

In the present study, 9 (13.0) patients had angina and/or ischemia according to ergometric test, 4 (44.4%) in the FFR group and 5 (55.6%) in the ANGIO group. In the ANGIO group, one patient was lost to follow-up before reassessment. All the four patients reassessed were treated for intra-stent stenosis defined by angiographic criteria, whereas in the FFR group, functional analysis indicated that 2 of these 4 patients required treatment. When we evaluated the need for new revascularization considering the presence of clinical restenosis (angina/ischemia) and functional reassessment, only half of patients in the FFR group was subjected to another PCI for intra-stent restenosis. In the ANGIO group, according to angiographic criteria, 12 vessels with restenosis were identified, which were later treated. In the FFR group, 8 vessels were reassessed, and only 2 required treatment. Thus, in the former group, the number of treated vessels was six times greater, with twice the number of TLR compared with the latter group.

These results contrast with those reported in the FAME study,^15^ probably because only PS (without inclusion of NPS) was used by the authors.

In addition, we could speculate that, considering the use of NPS in patients with multivessel diseases, the choice for FFR could provide additional benefit. Since the incidence of restenosis was higher in this population, although the percentages of lesions did not differ with the use of PS, there was a significant reduction in the total number of lesions, in absolute numbers, as described as follows:

**For NPS:**

**Situation 1:** considering a hypothetical restenosis rate of 20%, there will be 20 restenosis for every 100 lesions considered significant according to angiographic criteria.

**Situation 2:** for every 100 lesions functionally analyzed, 50 will be treated; considering the same hypothetical restenosis rate of 20%, there will be 10 restenosis.

**For PS:**

**Situation 1:** considering a hypothetical restenosis rate of 5%, there will be 5 restenosis for every 100 lesions considered significant according to angiographic criteria.

**Situation 2:** for every 100 lesions functionally analyzed, 50 will be treated; considering the same hypothetical restenosis rate of 5%, there will be 2.5 restenosis.

Thus, the use of functional analysis to determine the likelihood of recommending revascularization could prevent more restenosis (in absolute numbers) than NPS.

Considering TLR, only half of patients of the FFR group underwent another PCI, whereas in the ANGIO group, the number of vessels treated was six times greater and the need for TLR was twice higher. These findings differ from those reported in the FAME study,^15^ again, probably because only PS was used in their study.

Logistic regression of demographic, clinical and angiographical factors did not show increased risk for MACE, similar to the FAME study.^15^

### Angiographic data

In the FFR group, 45% of the lesions analyzed were treated, with a mean of 1.14 stent per patient; in the ANGIO group, all lesions were treated, with a mean of 2.2 stents per patient. The number of stents was 50% greater in the ANGIO group. In the FAME^15^ study, however, only 30% of the lesions were treated (2.7 stents per patient in the ANGIO group and 1.9 in the FFR group). The mean extension of stent coverage was 51.4 ± 2.0 mm and 37.9 ± 27.0 mm, respectively,^15^ and in our study we found a mean of 14.65 ± 6.91 mm. The mean FFR was 0.74 ± 0.15 mm in our study, very similar to that of the FAME study.^15^ Based on functional analysis, 55% and 37% of the lesions analyzed were not treated in the present study and in the FAME study,^15^ respectively; this difference may be due to the inclusion of more complex lesions treated by PS in our study. In addition, although mean stenosis percentage (60%) was similar in both studies, mean diameter of target vessel was greater in our study (2.9 ± 0.4 mm and 2,8 ± 0,5 mm in FFR and ANGIO groups, respectively) compared with the FAME study^15^ (mean of 2.5 mm in both groups).

### Cost-effectiveness

CE compares costs and effects of different health technologies to identify which technique provides the greatest benefit, and the incremental cost (IC) for it. In this economic analysis, costs are expressed in monetary units, whereas effects in clinical-epidemiological units or natural units (prevented cases, survival, cure, etc.). The main of CE analysis is to maximize the outcomes in health with the financial resources available. The most common outcome measure of CE analysis is ICER, which represents the ratio between costs of the techniques (cost of A - cost of B) and effectiveness of the techniques (effectiveness of A - effectiveness of B). This ratio is used to identify which of these strategies result in maximal effectiveness for a given cost, or the degree of investment required to obtain incremental benefit in health.

CE criterion is one of many criteria that should be used to determine whether an intervention should be offered. In addition, equity, needs and priorities should also be considered in the decision-making process. CE relates costs with clinical outcomes and compare relative value of interventions; it translates the difference of costs between two strategies of treatment. The monetary value is divided by the difference of their effectiveness, expressed in years of life gained (life expectancy) or other prevented or avoided events.^21^

Quality-adjusted life year (QALY) is a measure of disease burden, of both quality and quantity of life. QALY is used to evaluate the cost-benefit ratio of a therapeutic intervention.^21^ In monetary values, therapies with costs lower than USD20,000/QALY are considered favorable strategies; those with costs from USD20,000 to USD40,000/QALY are consistent with habitual interventions, and therapies with costs higher than USD40,000/QALY are considered of little benefit.

CE of an intervention is known to vary with overall individual or population risk;^21^ however, in Brazil, the incremental costs of an intervention that provide clinical benefits have not been established. In both American and Canadian health systems, the value of USD50,000 per QALY, and more recently USD10,000 per prevented major event is considered a reasonable utilization of health resources.

In the present study, the difference of effectiveness in one year was 1.82%; however, ICER, established as the difference of costs between PCI in the ANGIO group and PCI in the FFR group divided by the difference in effectiveness (one-year-restenosis-free survival) was BRL21,156.55. This value is consistent with optimal therapies as well as with overall individual or population risk, and therefore considered cost-effective.

We did not find in the literature studies on the CE of FFR-guided PCI and NPS in patients with multivessel diseases, which is hence a strength of our study. Our findings demonstrate clinical benefits of CE during one year of follow-up, which is not commonly seen in new therapeutic strategies, as shown by Fearon et al.,^22^ suggesting an economic or social impact. The use of FFR in PCI in multivessel disease patients is a more cost-effective approach than treating all significant lesions identified by angiography. This can help change the paradigm and reduce costs^23^ at the same time and thereby consolidate the practice of medicine based on physiological data, which would lead to better medical care.

### Study limitations

The sample size was small, particularly due to limited funding resources, which made it difficult to obtain more consistent clinical data. Despite that, we did show significant differences in CE and reduction in TLR.

Due to the long period of patient recruitment, some multivessel disease patients treated by angioplasty could not be recruited because of logistic and financial issues.

## Conclusions

FFR-guided PCI, as compared with angiographic criteria,is a cost-effective strategy that reduces the number of lesions treated, stents, and the need for TVR in patients with multivessel diseases.

## References

[1] Hachamovitch R, Rozanski A, Stone GW, Thomson LE, Friedman JD (2011). Impact of ischaemia and scar on the therapeutic benefit derived
from myocardial revascularization vs. medical therapy among patients
undergoing stress-rest myocardial perfusion scintigraphy. Eur Heart J..

[2] Aarnoudse WH, Botman KJ, Pijls NH. (2003). False-negative myocardial scintigraphy in balanced three-vessel
disease, revealed by coronary pressure measurement. Int J Cardiovasc Intervent.

[3] Fischer JJ, Samady H, McPherson JA, Sarembock IJ, Gimple LW (2002). Comparison between visual assessment and quantitative angiography
versus fractional flow reserve for native coronary narrowings of moderate
severity. Am J Cardiol.

[4] De Bruyne B, Fearon WF, Pijls NH, Barbato E, Tonino P, Piroth Z (2014). FAME 2 Trial Investigators. Fractional flow reserve-guided PCI
for stable coronary artery disease. N Engl J Med.

[5] Sant’Anna FM, Silva ER, Batista LA, Brito MB (2008). Qual o erro da angiografia na definição de isquemia
miocárdica durante intervenções coronarianas
percutâneas?. Arq Bras Cardiol.

[6] Pijls NH. (2004). Optimum guidance of complex PCI by coronary pressure
measurement. Heart.

[7] Sant’Anna FM, Silva EE, Batista LA, Ventura FM, Barrozo CA, Pijls NH. (2007). Influence of routine assessment of fractional flow reserve on
decision making during coronary interventions. Am J Cardiol.

[8] Pijls NH, van Schaardenburgh P, Boersma E, Bech JW, van’t Veer M (2007). Percutaneous coronary intervention of functionally nonsignificant
stenosis: 5-year follow-up of the DEFER Study. J Am Coll Cardiol.

[9] Fearon WF, Yeung AC, Lee DP, Yock PG, Heidenreich PA. (2003). Cost-effectiveness of measuring fractional flow reserve to guide
coronary interventions. Am Heart J.

[10] Polanczyk CA, Wainstein MV, Ribeiro JP. (2007). Cost-effectiveness of sirolimus-eluting stents in percutaneous
coronary interventions in Brazil. Arq Bras Cardiol.

[11] Hachamovitch R, Kang X, Amanullah AM, Abidov A, Hayes SW, Friedman JD (2009). Prognostic implications of myocardial perfusion single-photon
emission computed tomography in the elderly. Circulation.

[12] Shaw LJ, Berman DS, Maron DJ, Hartigan PM, COURAGE Investigators (2008). Optimal medical therapy with or without percutaneous coronary
intervention to reduce ischemic burden: results from the Clinical Outcomes
Utilizing Revascularization and Aggressive Drug Evaluation (COURAGE) trial
nuclear substudy. Circulation.

[13] International Study of Comparative Health Effectiveness with Medical
and Invasive Approaches Executive summary of the ISCHEMIA trial 2013.

[14] Brasil, Ministério da Saúde, Secretaria de Ciência, Tecnologia e Insumos
Estratégicos, Departamento de Gestão e Incorporação de
Tecnologias em Saúde (2014). Relatório de Recomendação da Comissão
Nacional de Incorporação de Tecnologias no SUS - CONITEC -
111. Stent farmacológico para o tratamento da DAC.

[15] Tonino PA, Fearon WF, De Bruyne B, Oldroyd KG, Leesar MA, Ver Lee PN (2010). Angiographic versus functional severity of coronary artery
stenoses in the FAME study: fractional flow reserve versus angiography in
multivessel evaluation. J Am Coll Cardiol.

[16] Bravata DM, Gienger AL, McDonald KM, Sundaram V, Perez MV, Varghese R (2007). Systematic review: the comparative effectiveness of percutaneous
coronary interventions and coronary artery bypass graft
surgery. Ann Intern Med.

[17] Li J, Elrashidi MY, Flammer AJ, Lennon RJ, Bell MR, Holmes DR (2013). Longterm outcomes of fractional flow reserve-guided vs.
angiography-guided percutaneous coronary intervention in contemporary
practice. Eur Heart J..

[18] De Backer O, Biasco L, Lønborg J, Pedersen F, Holmvang L, Kelbaek H (2016). Long-term outcome of FFR-guided PCI for stable coronary artery
disease in daily clinical practice: a propensity score-matched landmark
analysis. EuroIntervention.

[19] Van Belle E, Rioufol G, Pouillot C, Cuisset T, Bougrini K, Teiger E (2014). Investigators of the Registre Français de la FFR-R3F.
Outcome impact of coronary revascularization strategy reclassification with
fractional flow reserve at time of diagnostic angiography: insights from a
large French multicenter fractional flow reserve registry. Circulation.

[20] Zhang D, Lv S, Song X, Yuan F, Xu F, Zhang M (2015). Fractional flow reserve versus angiography for guiding
percutaneous coronary intervention: a metaanalysis. Heart.

[21] Moreira PBB. (2004). Custo-efetividade de programas de reabilitação
cardiovascular. Jornal do Departamento de Ergometria e Reabilitação
Cardíaca.

[22] Fearon WF, Bornschein B, Tonino PA, Gothe RM, Bruyne BD, Pijls NH (2010). Fractional Flow Reserve Versus Angiography for Multivessel
Evaluation (FAME) Study Investigators. Economic evaluation of fractional
flow reserveguided percutaneous coronary intervention in patients with
multivessel disease. Circulation.

[23] Teich V, Araújo DV. (2011). Estimativa de custo da síndrome coronariana aguda no
Brasil. Rev Bras Cardiol.

